# Innovative Pulp Preservation: The Use of Platelet-Rich Fibrin (PRF) and Mineral Trioxide Aggregate (MTA) in Treating Dental Pulp Exposure

**DOI:** 10.7759/cureus.63740

**Published:** 2024-07-03

**Authors:** Palak Hirani, Swayangprabha Sarangi, Manoj Chandak, Aditya Patel, Anuja Ikhar, Kapil Naladkar

**Affiliations:** 1 Department of Conservative Dentistry and Endodontics, Sharad Pawar Dental College, Datta Meghe Institute of Higher Education and Research, Wardha, IND

**Keywords:** dentin regeneration, platelet rich fibrin, vital pulp therapy, mineral trioxide aggregate [mta], pulp vitality, biomimetics

## Abstract

The dental pulp, essential for tooth vitality, often becomes inflamed when exposed due to caries, fractures, or dislodged restorations. Untreated inflammation can lead to pulpal death, necessitating vital pulp therapies (VPTs) such as pulp capping and pulpotomy. Recent trends favor partial caries removal to avoid overtreatment and preserve pulp health. This shift is illustrated through two cases of young female patients with dislodged restorations and deep caries. Both underwent direct pulp capping using platelet-rich fibrin (PRF) and mineral trioxide aggregate (MTA), followed by composite restorations. These cases underscore the importance of biocompatible materials like MTA and PRF in maintaining pulp vitality and promoting dental tissue repair.

## Introduction

Vital to the tooth's survival, the dental pulp is a thin layer of connective tissue encased in the tough dentin walls. Inflammation of the pulp is common when it is exposed due to fractures, cracks, cavities, or an exposed restorative margin. Pulpal death could result if this inflammation is not treated immediately [1]. A conservative treatment method for teeth with pulp tissue weakened by trauma, dental caries, or restorative operations is called vital pulp therapy. Dental pulp diseases can be treated using essential pulp therapies, which include pulpectomy if the lesion appears later on, pulpotomy if the lesion appears early, and direct and indirect pulp capping. Preserving pulpal vitality is essential because a sound, functioning pulp can initiate several key processes, such as dentin synthesis, tooth nourishment, defense, and a unique capacity for restoration. Thus, it is better to preserve the pulp's vitality rather than using an inert root filler in its place [2].

Long-term tooth viability depends critically on the preservation of pulp vitality. Ricketts et al. state that the existence of a profound carious lesion and the treatment that follows can damage the vitality of the tooth pulp. Traditionally, complete (non-selective) caries removal has been used to treat these lesions; however, new research indicates that more cautious partial (selective) caries removal techniques may be able to minimize the pulp exposure risk related to total caries removal. Non-selective caries removal techniques are still widely used despite advances in our knowledge of the advantages of selective caries removal for the management of deeply embedded carious lesions. Thus, Hoefler et al. state that even when a selective caries removal method is used in clinical practice, carious exposure in deep lesions can still happen in cases when there are little to no symptoms [3].

Indirect pulp capping is a vital pulp therapy procedure where only the infected dentin is removed, leaving a thin layer of affected dentin further to line the cavity floor with suitable capping material and ensure a complete seal against microleakage with an intact restoration. This ensures complete lesion sterilization and tissue regeneration. Depending on the case selection and clinical necessity, either a one-step or two-step process is used to accomplish indirect pulp capping. According to Brodén et al. (2016), when it came to young permanent teeth with carious exposures, the clinical and radiographic success of direct pulp capping (DPC) was high. Additionally, Brodén et al. 2019 modeling studies have suggested that DPC is a more affordable option for treating carious exposures in children and young adults than root canal therapy [4].

It has been recommended that biologically-based treatment techniques for partial caries eradication be promoted in order to prevent exposure to carious pulp. In fact, the total or nonselective removal of carious material is currently regarded as overtreatment, according to recent consensus assessments [3]. As pulpectomy is avoided in favor of vital pulp treatment (VPT) methods, including pulp capping and partial and complete pulpotomy, management tactics for the treatment of the cariously exposed pulp are also changing [5].

In 1993, mineral trioxide aggregate (MTA) was introduced by altering Portland cement. Tricalcium silicate, bismuth oxide, tricalcium oxide, tricalcium aluminate, and silicate oxide make up MTA. MTA is a pulp-capping substance based on calcium silicate. MTA stimulates cytokines and growth factors to start the production of reparative dentin. MTA has superior dentin marginal sealing [6]. The use of this material as an excellent pulp capping material is limited by its longer setting time, which is a possibility for staining teeth and handling problems [7].

Biodentine (Septodont, Saint-Maur-des-Fossés, France) was introduced in 2009, a silicate-based cement with outstanding qualities. Tricalcium silicate, zirconium oxide, and calcium carbonate are combined to create a powder. Calcium chloride is added to the liquid as a water-reducing agent and setting accelerator. It does not stain teeth when it first sets, which takes 12 minutes. Biodentine stimulated the production of reparative dentin without inflaming the pulp [8].

## Case presentation

Case presentation - 01

A 17-year-old female patient reported to the Department of Conservative Dentistry and Endodontics and complained of dislodged restoration and pain in the lower left back region of the jaw for one month. There was no pertinent medical history available. An intraoral examination revealed dislodged restoration with deep occlusal caries, which was seen in 36. Pit caries were observed in 16, 46, 17, and 47. Tenderness on percussion negative with 36. No mobility or depressibility with 36. On electric pulp testing (EPT), 36 showed a similar response when compared to the contralateral tooth (46).

**Figure 1:**
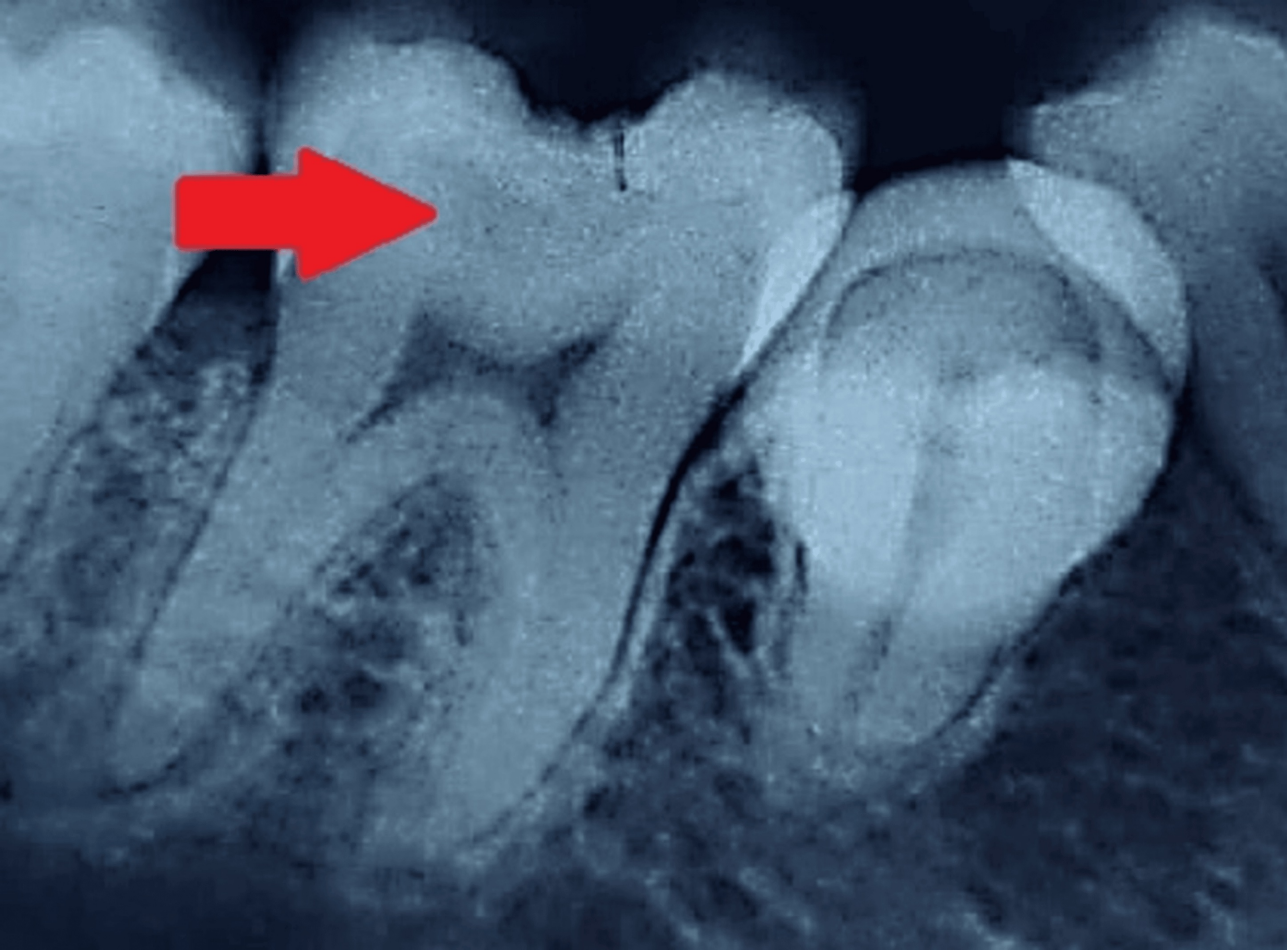
Preoperative intra-oral periapical radiograph showing deep carious lesion in the lower right mandibular first molar The arrow shows radiolucency involving enamel, dentin, and approaching pulp.

On radiographic examination, radiolucency was found involving enamel, dentin, and approaching pulp along with PDL widening, which was evident with 36, as shown in Figure 1.

Treatment

**Figure 2:**
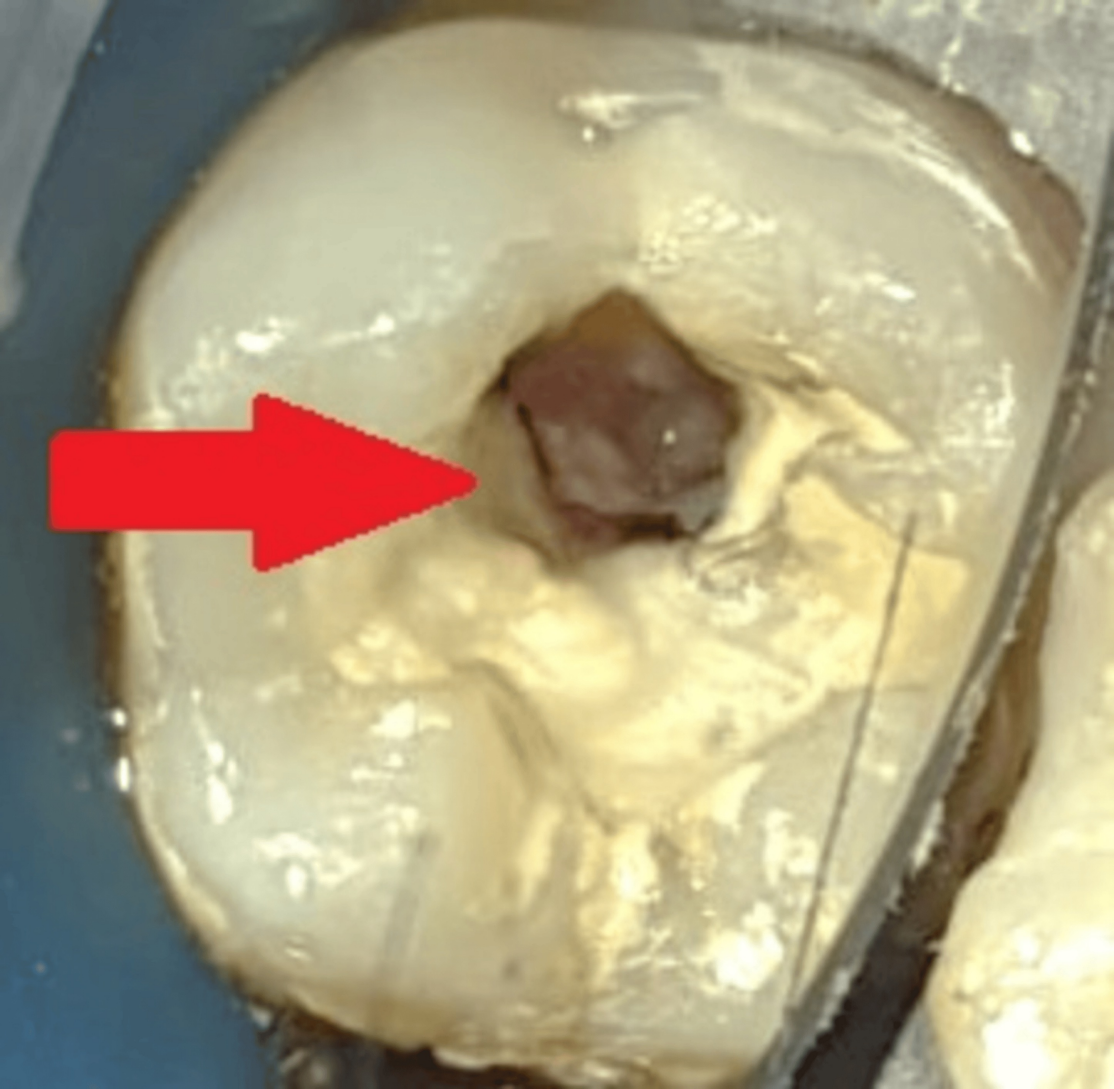
Preoperative clinical view under rubber dam isolation The arrow shows a clinical picture of a carious lesion.

The patient was informed that DPC - a treatment option that replaces traditional root canal therapy - uses platelet-rich fibrin (PRF) and MTA, a cement with a calcium silicate basis. The patient's signed consent was acquired. After a medical examination and tests, the results showed that the patient's bleeding, clotting, and platelet counts were within normal limits. Lower left back inferior alveolar nerve block (2% lignocaine 1:2,00,000) was used. A rubber dam was used to isolate the teeth (GDC Dental Dam Kit, Hoshiarpur, India), as shown in Figure 2.

**Figure 3:**
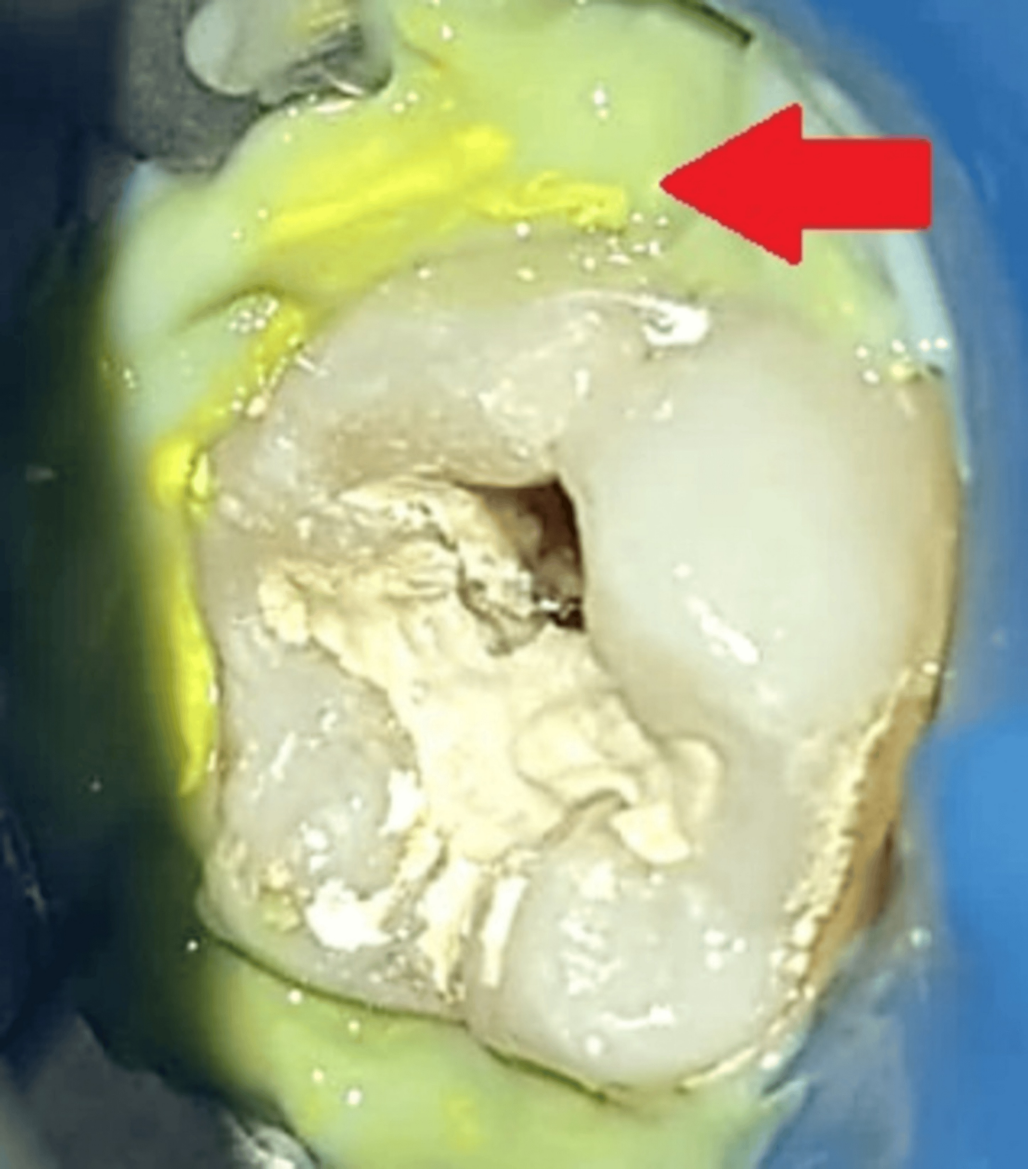
Clinical picture after gingidam application The arrow shows gingidam application.

Since the rubber dam was lingually inserted, total isolation of the tooth was not accomplished after it was placed; hence, additional gingidam application was necessary, as shown in Figure 3.

**Figure 4:**
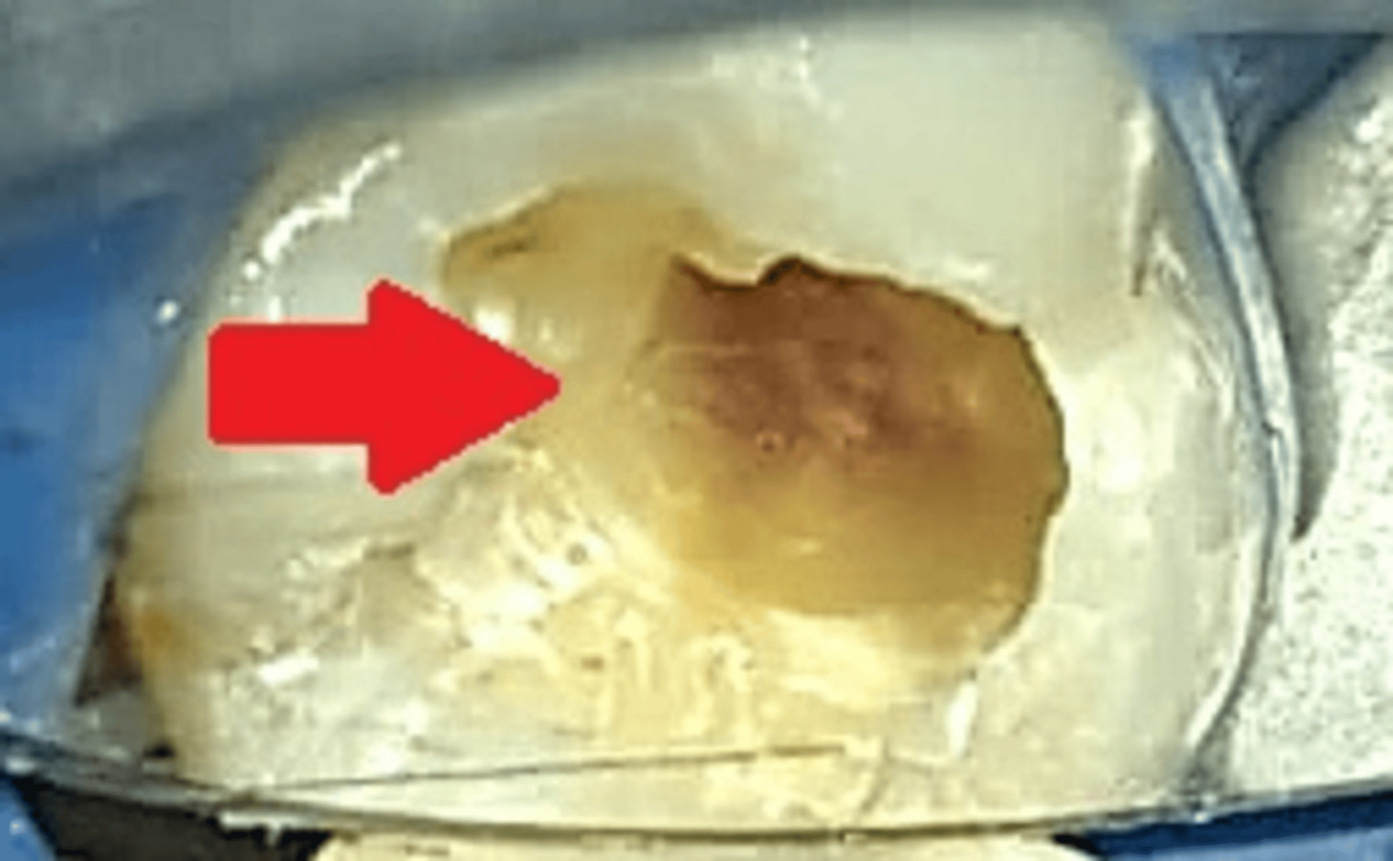
Clinical picture after removal of coronal caries The arrow shows the removal of caries.

After gaining access to the carious lesion, a high-speed air rotor handpiece with a round diamond point (BR 41; MANI, Tochigi, Japan) was used to remove coronal caries, as shown in Figure 4.

**Figure 5:**
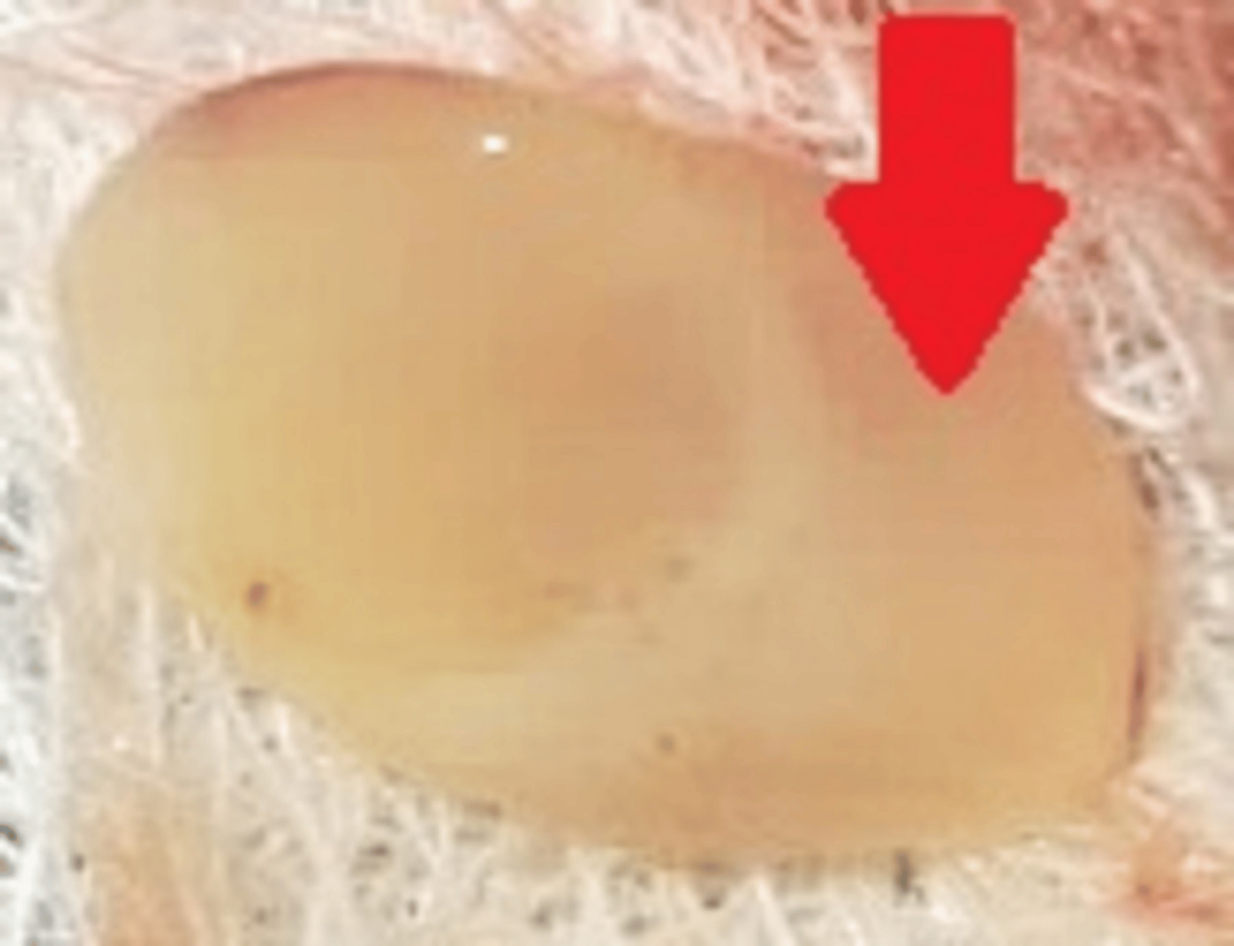
Platelet-rich fibrin The arrow shows platelet-rich fibrin.

Spoon excavators were used to remove soft, carious dentin from the periphery. After the carious dentin was removed, a single bleeding point was visible on the distal side of the hollow surface that was exposed. Pressure was applied to cotton pellets that had been wet with saline, and hemostasis was achieved; 2% chlorhexidine (CHX) was used to sterilize the cavity. Over the exposed pulp stumps, the PRF membrane was applied following the centrifugation of the patient's own blood extracted from the median cubital vein, as shown in Figure 5.

**Figure 6:**
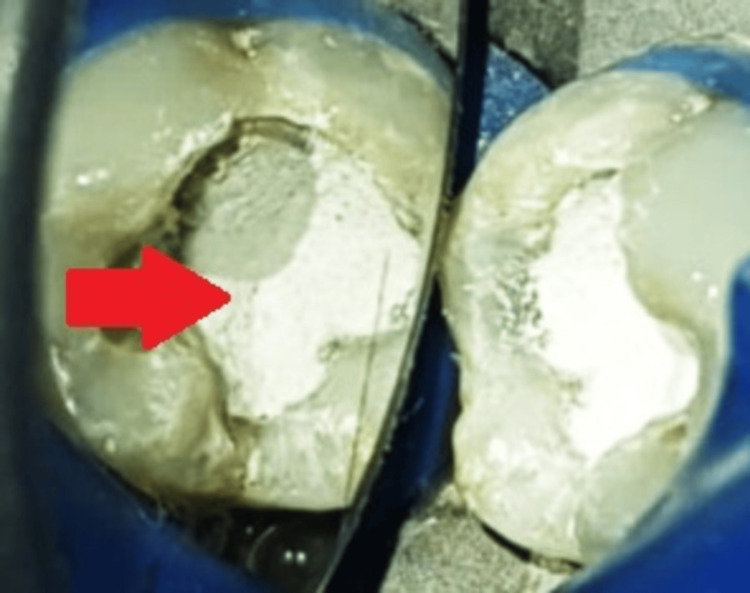
Placement of MTA Angelus over PRF The arrow shows MTA placed over PRF. MTA - mineral trioxide aggregate; PRF - platelet-rich fibrin

After placing MTA Angelus (Angelus, Londrina, Brazil) directly on top of the PRF membrane, it was moistened with a cotton pellet, as shown in Figure 6. 

**Figure 7:**
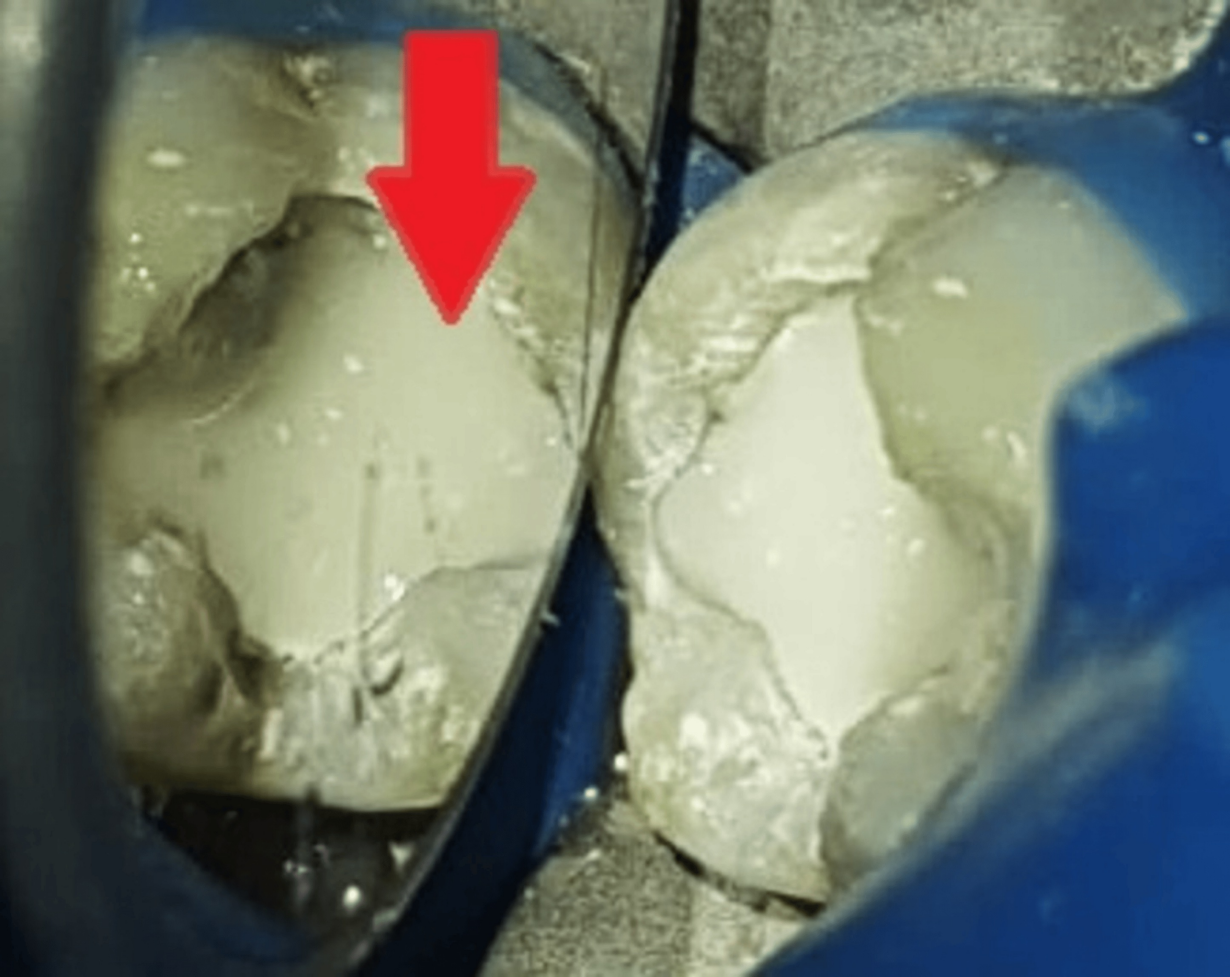
Flowable glass ionomer cement The arrow shows the placement of flowable glass ionomer cement (GIC).

The set mass of MTA was then covered with flowable glass ionomer cement (RMGIC; GC Fuji II LC®, Tokyo, Japan), as shown in Figure 7.

**Figure 8:**
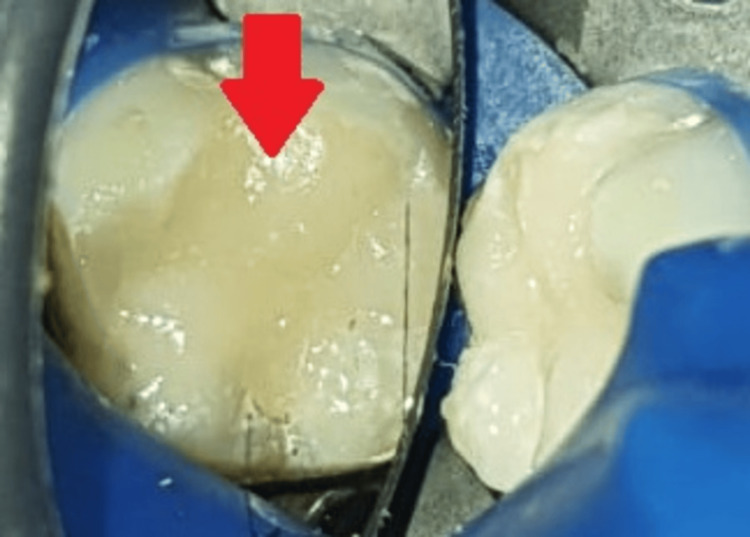
Direct composite placement The arrow shows direct composite permanent restoration.

Direct composite (Tetric-Ceram; Ivoclar Vivadent Inc., Amherst, USA) restoration was placed on 36, as shown in Figure 8.

**Figure 9:**
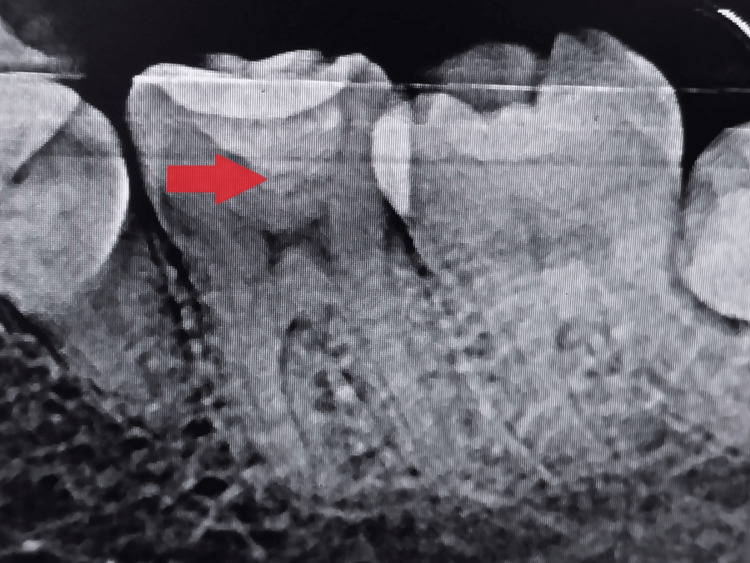
Follow-up intraoral periapical radiograph after six months The arrow shows the placement of MTA followed by GIC and a direct composite as a permanent restoration. GIC - glass ionomer cement; MTA - mineral trioxide aggregate

**Figure 10:**
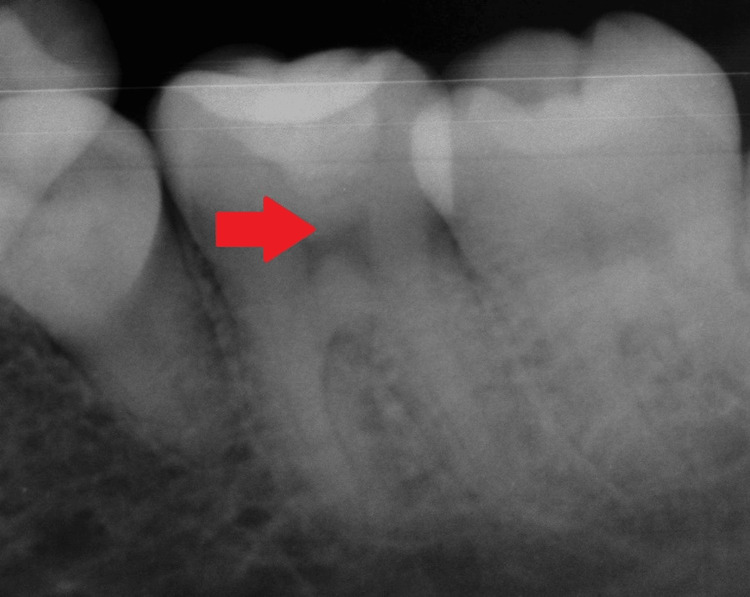
Follow-up intraoral periapical radiograph after one year The arrow shows the formation of the dentin bridge at a one-year follow-up.

Further follow-ups at three months and six months are shown in Figure 9 and Figure 10, respectively.

Case presentation - 02

A 22-year-old female patient reported to the Department of Conservative Dentistry and Endodontics and complained of food lodgment in the jaw's lower right back area for a month. The patient was apparently all right one month back when she started experiencing food lodgment in the lower right back region of the jaw, which was associated with mild pain and was intermittent in nature. The pain was non-lingering and was not associated with swelling. There was no pertinent medical history available. Past dental history showed a history of restoration at 46. An intraoral examination revealed a dislodged restoration with secondary caries in 46 (tenderness on percussion was negative). Pit caries were observed in 26 and 36. EPT for 46 showed an early response when compared to the contralateral tooth (36).

**Figure 11:**
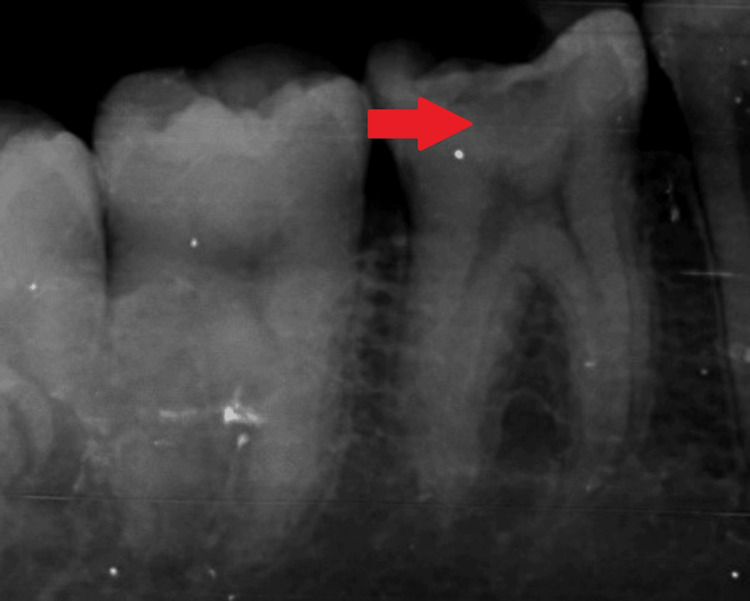
Preoperative intraoral periapical radiograph The arrow shows radiolucency involving enamel, dentin, and approaching pulp.

On radiographic examination, radiolucency was seen on the occlusal surface with 46 involving enamel, dentin, and approaching pulp. No periapical changes were seen, as shown in Figure 11.

Treatment

**Figure 12:**
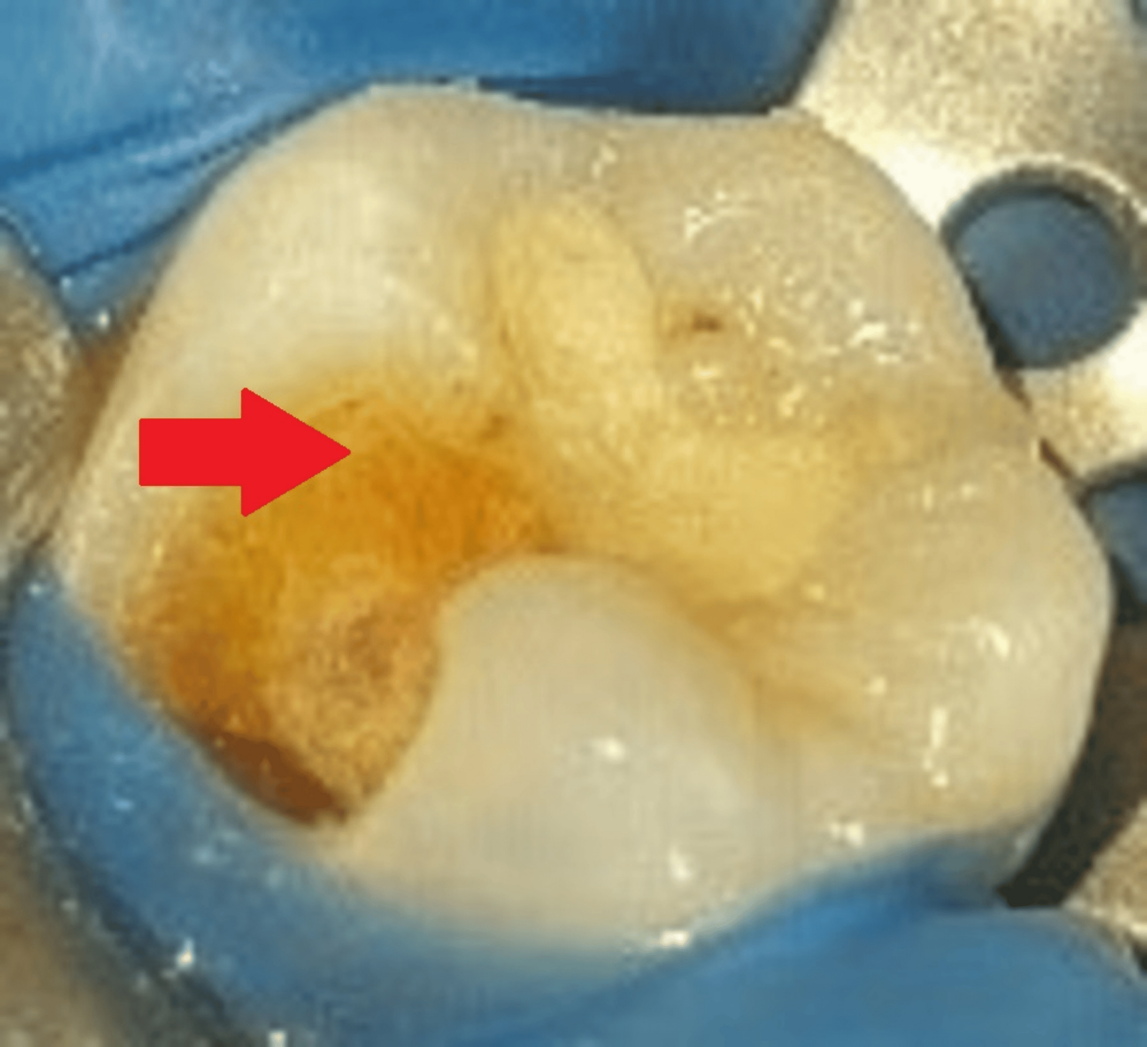
Preoperative clinical view The arrow shows a clinical picture of a carious lesion.

Lower left back inferior alveolar nerve block (2% lignocaine 1:2,00,000) was given. Rubber dam isolations were completed as shown in Figure 12.

**Figure 13:**
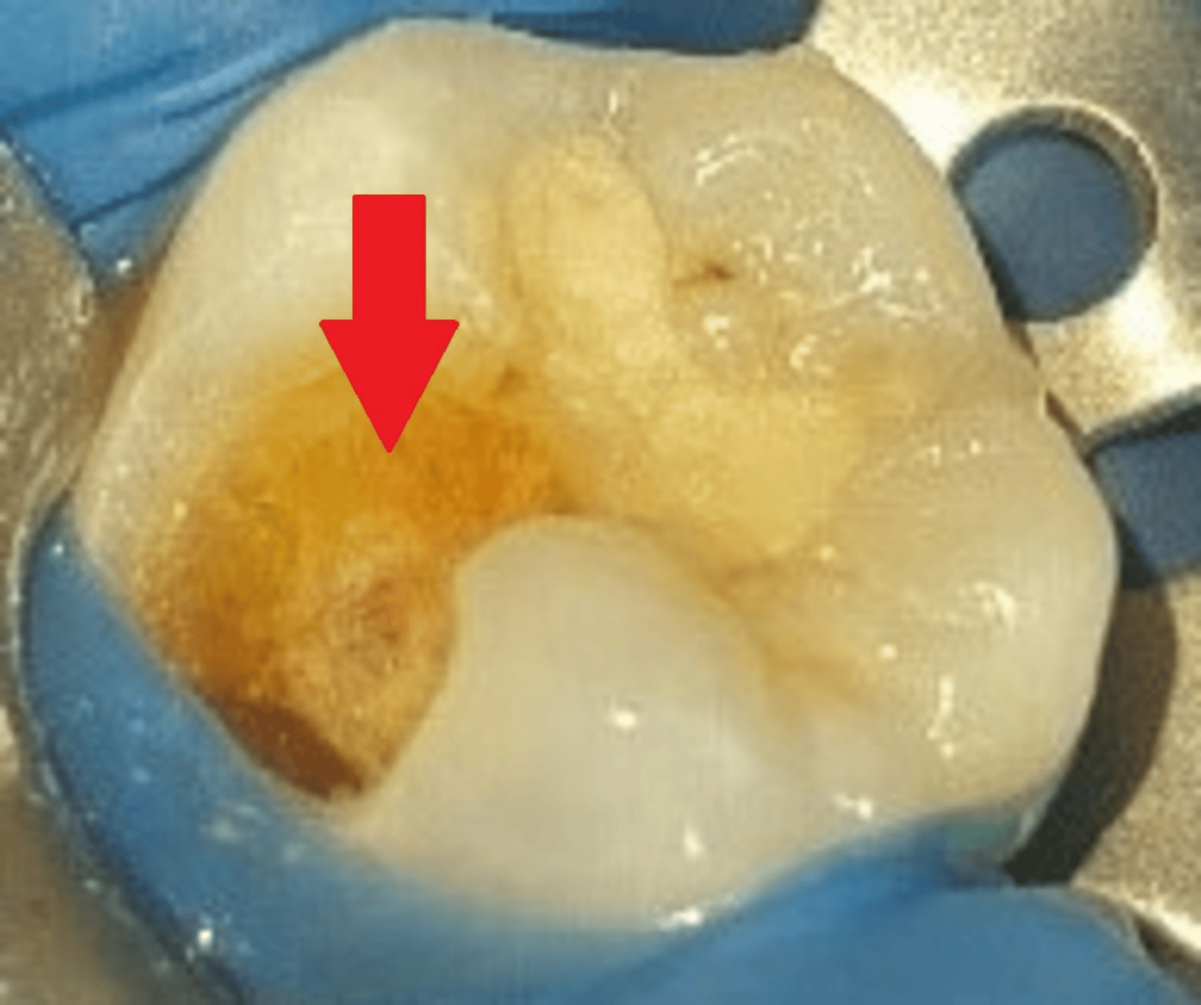
Removal of infected caries using round bur The arrow shows the removal of infected caries.

Removal of infected dentin from 46 was performed using a round diamond point, as shown in Figure 13.

**Figure 14:**
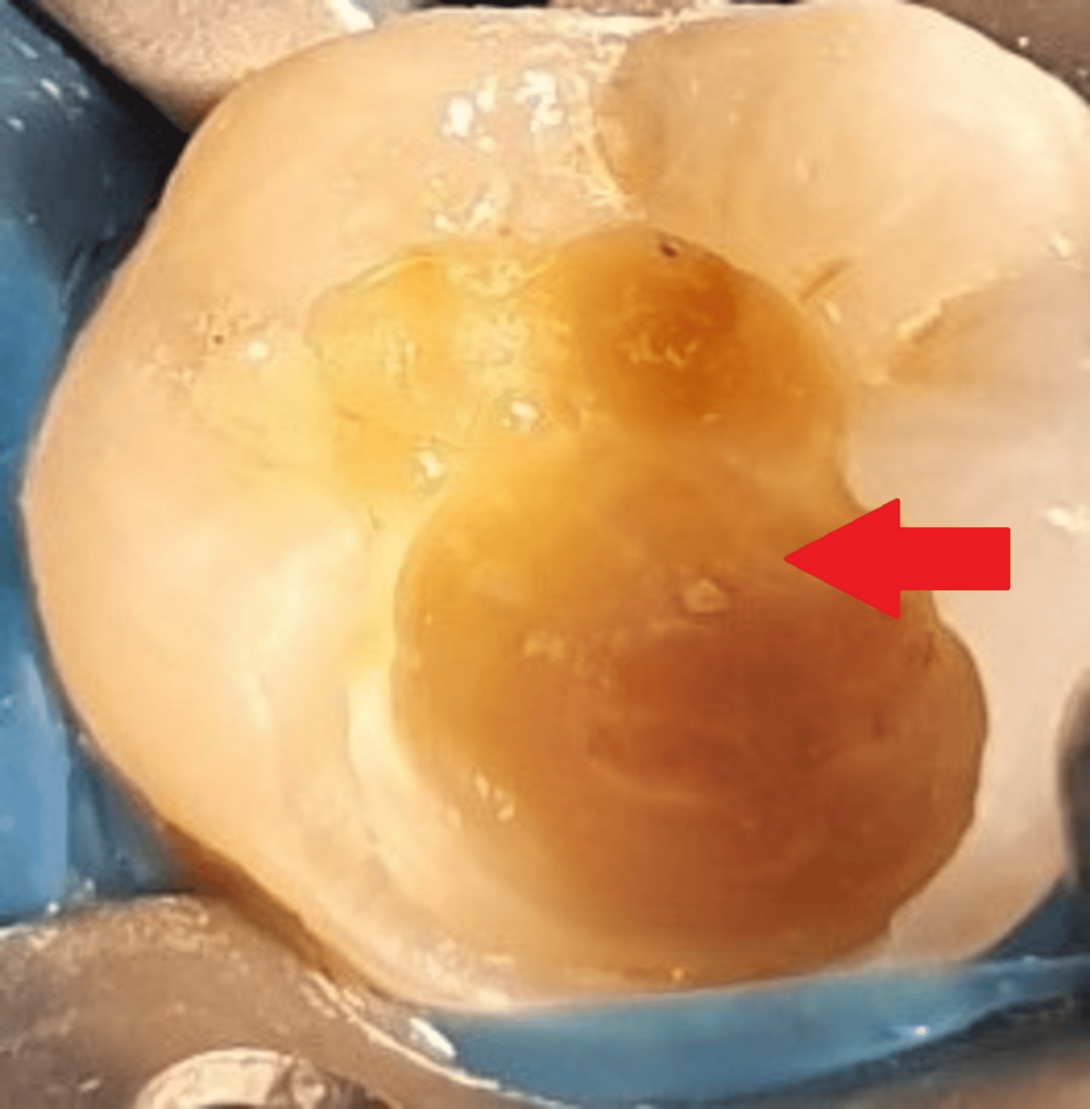
Removal of peripheral caries dentin The arrow shows the removal of peripheral carious dentin.

A spoon excavator was used to remove peripheral carious dentin, as shown in Figure 14.

**Figure 15:**
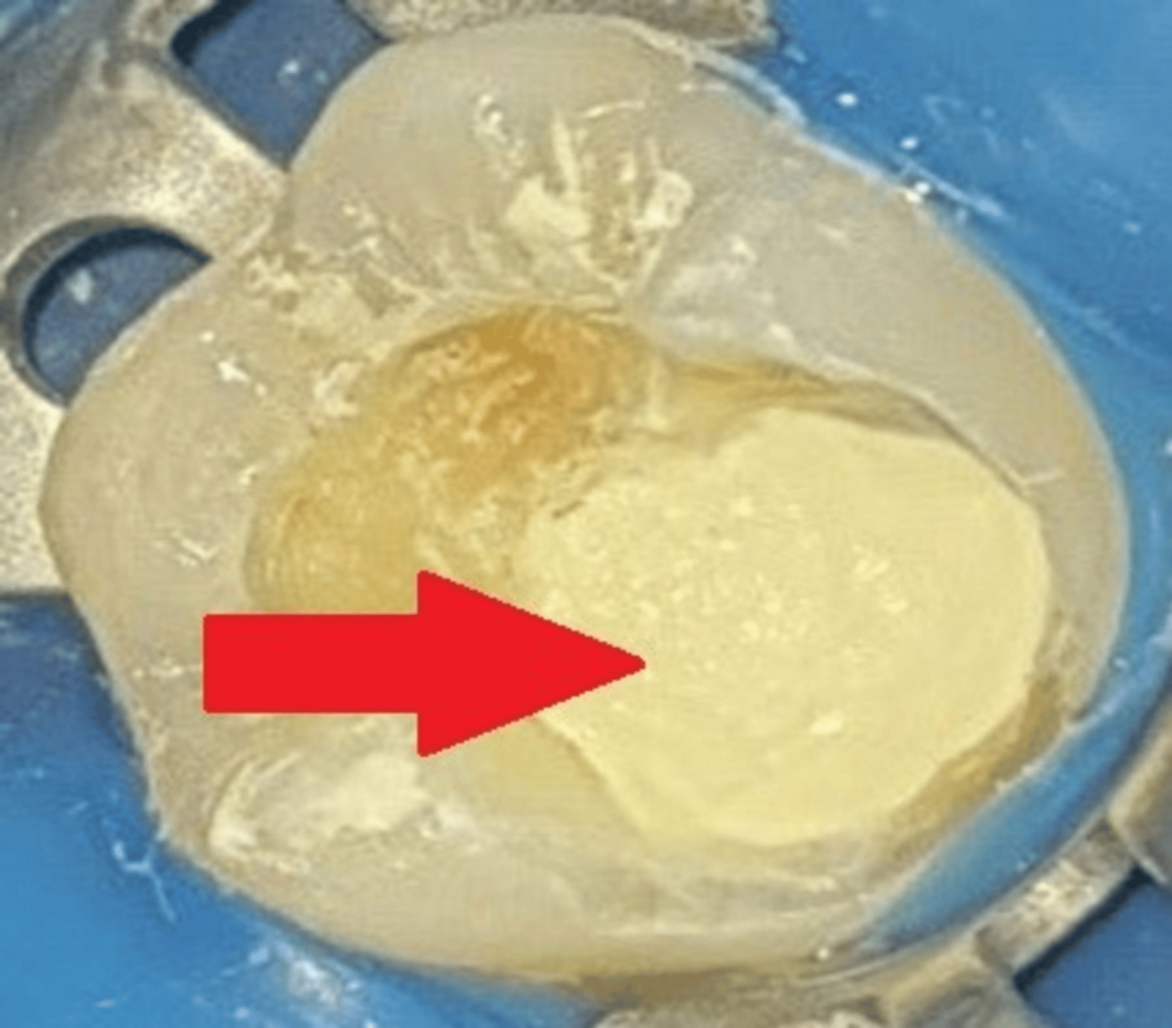
Clinical picture of placement of MTA The arrow shows the placement of the MTA. MTA - mineral trioxide aggregate

The cavity was cleaned for 60 seconds with 2% CHX, and MTA Angelus was placed, as shown in Figure 15.

**Figure 16:**
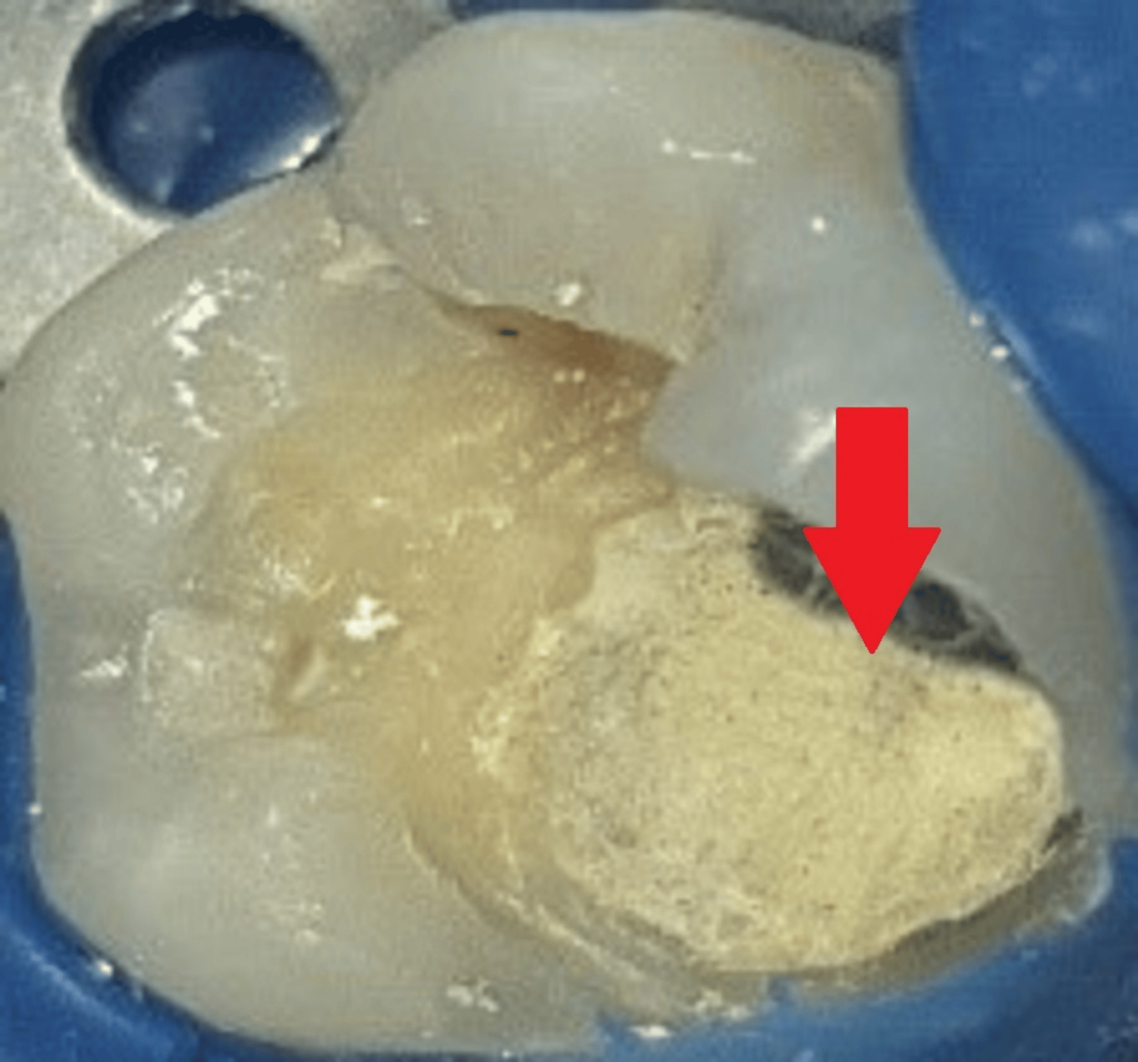
Removal of temporary restoration and damp cotton The arrow shows clinical picture after removal of temporary restoration and damp cotton at the second visit.

Removal of the temporary restoration and the damp cotton from 46 was performed at the second appointment, as shown in Figure 16.

**Figure 17:**
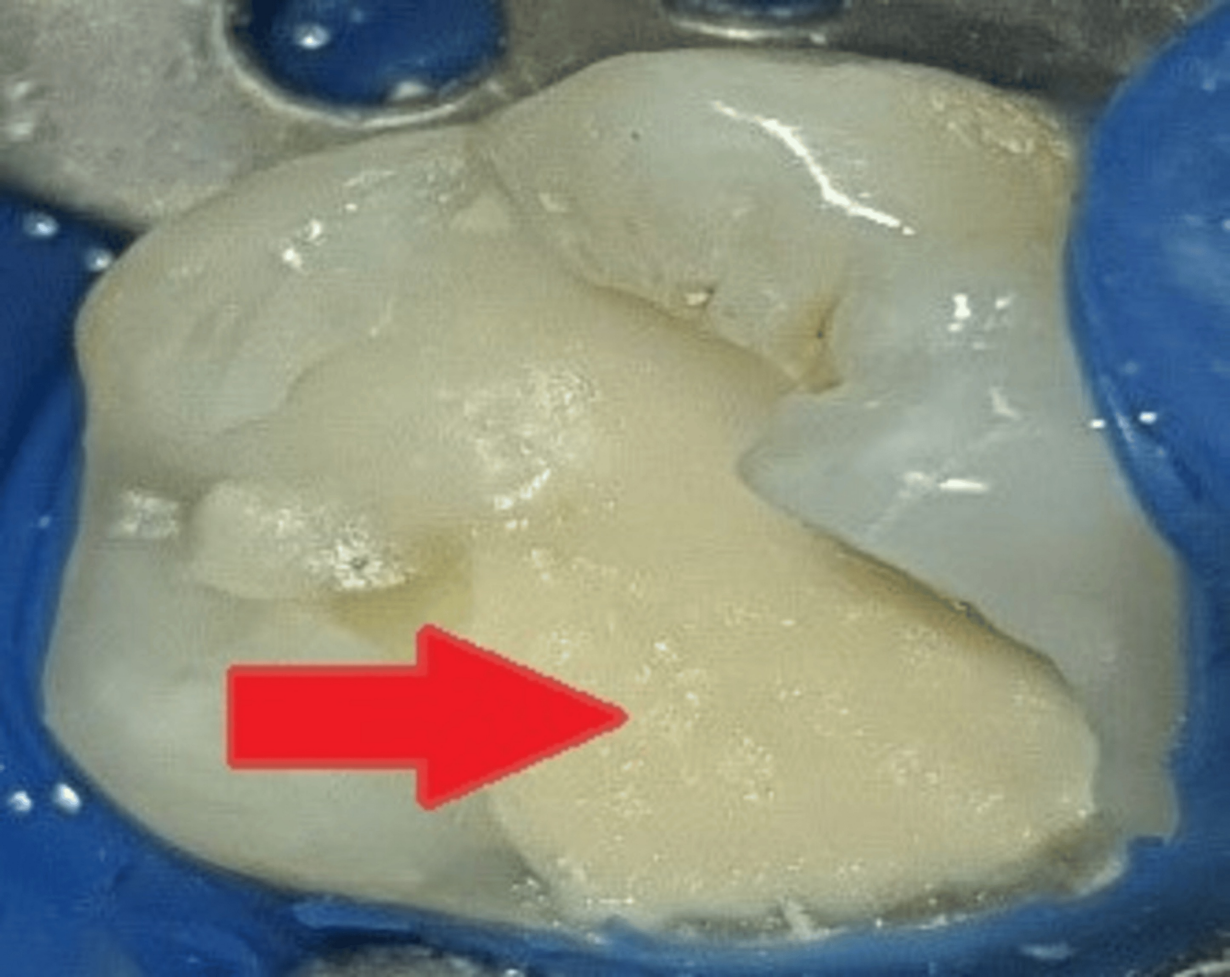
Placement of flowable GIC The arrow shows flowable GIC. GIC - glass ionomer cement

The light-cure base of glass ionomer cement was set up on 46, as shown in Figure 17.

**Figure 18:**
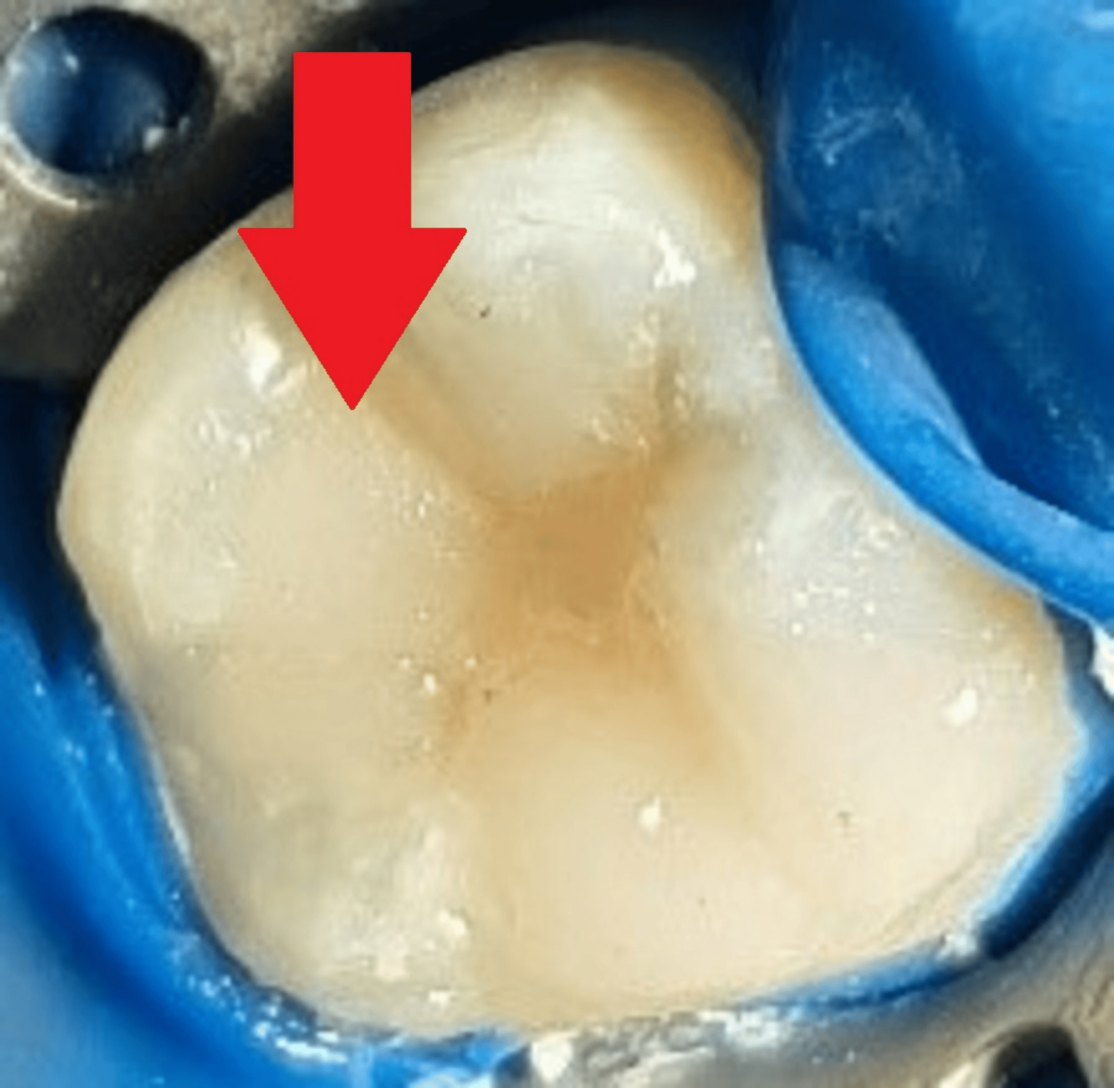
Clinical picture of permanent direct composite restoration The arrow shows direct composite permanent restoration.

This was followed by a composite restoration on 46, as shown in Figure 18.

**Figure 19:**
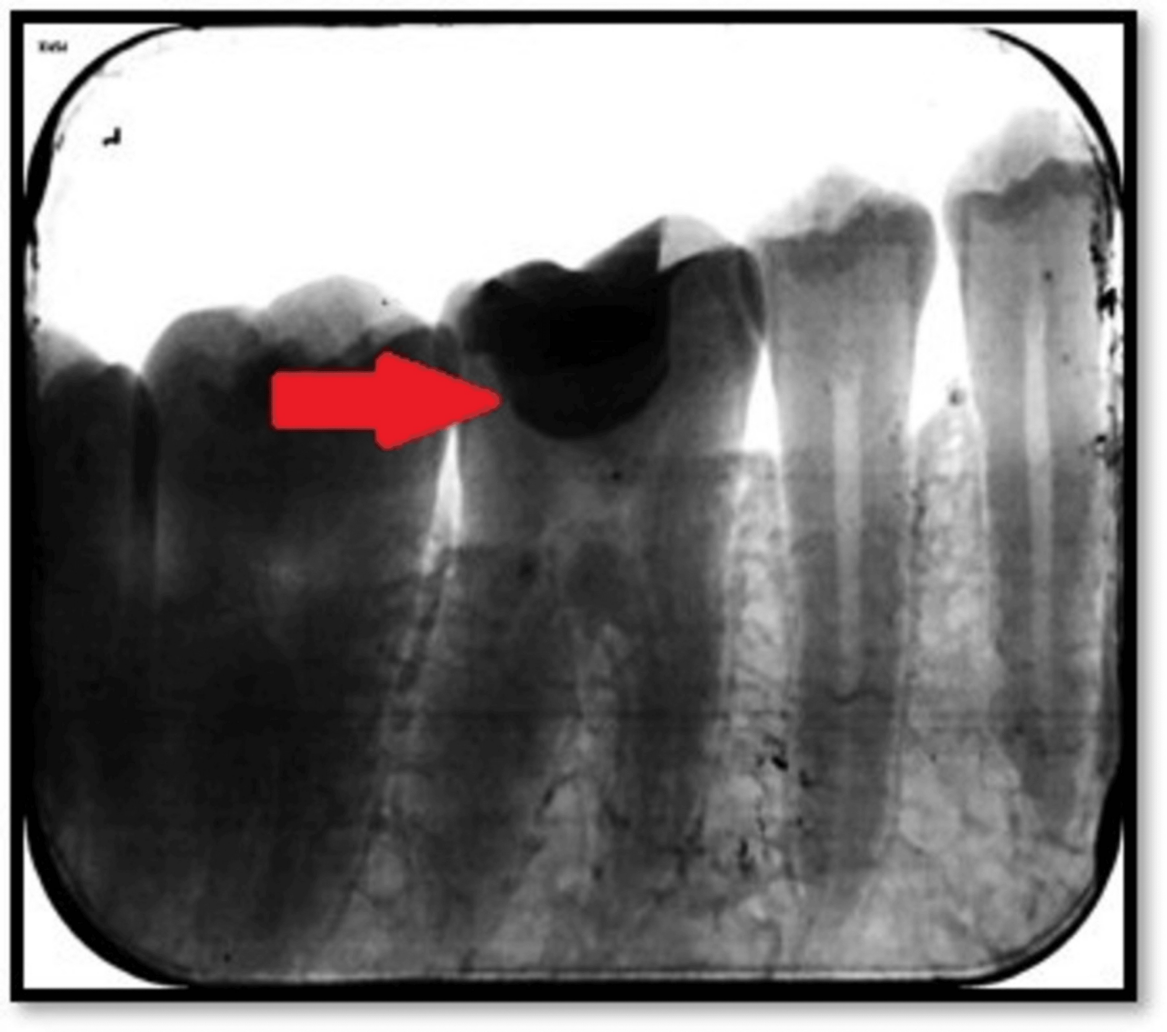
Immediate postoperative intraoral periapical radiograph The arrow here denotes the condensed mineral trioxide aggregate followed by a GIC base and a permanent composite restoration. GIC - glass ionomer cement

An immediate postoperative radiograph is shown in Figure 19.

The patient did not report to the department for further follow-up.

## Discussion

Calcium hydroxide, MTA, and biodentine are the ingredients used for DPC the most frequently. Hermann found that an exposed site could be effectively repaired with calcium hydroxide in 1930. Since then, clinical trials have successfully employed calcium hydroxide in the form of powder, paste, and cement to promote the development of reparative dentin while preserving essential pulp. Zinc oxide eugenol was first introduced by Glass and his associates for DPC [3]. However, there was no dentin bridge creation, only persistent inflammation and poor pulp repair. Eugenol was eventually shown to be extremely poisonous, and eugenol with zinc oxide caused a significant interfacial leakage. Glucocorticoids and antibiotics were often administered in the 1970s in an effort to reduce pulpal inflammation and regulate pulpal discomfort. Steroids are no longer utilized for DPC due to reports of poor wound healing and even pulpal necrosis [2].

Because MTA is more biocompatible and has better sealing capabilities than calcium hydroxide, it is regarded as a trustworthy substitute for this chemical in critical pulp therapy procedures. It offers superior marginal adaptability and has outstanding physical qualities [1]. Compared to calcium hydroxide cement, it promotes reparative dentin production more quickly and keeps a high pH for extended periods of time. Platelet-rich plasma (PRP) is a patient's restricted volume of plasma that has been enhanced with platelets. The research has shown that PRP can be used as a potentially perfect scaffold for regenerative endodontic therapy [5]. The controversy surrounding PRF, a second-generation, totally autologous platelet concentrate, was developed by Choukroun's PRP activation using bovine thrombin. Choukroun et al. created PRF in France in 2001. This is a very low-cost, easy procedure.

Platelets, growth factors, and cytokines found in PRF may improve the ability of both soft and hard tissues to repair [3]. Titanium possesses strong hemocompatibility and resistance to corrosion, which are crucial characteristics for biomaterials that interact with blood. The stimulation of platelets was carried out in an identical fashion in both titanium and glass tubes. The development of clots in the titanium tubes was shown to be clinically equivalent to that of clots in the glass tubes. The fibrin structure of titanium-PRF seemed thicker and more tightly intertwined [2]. The titanium fibrin carpet has a more resilient network structure. To prolong the time it takes for fibrin to resorb and lengthen the time it takes for growth factors to be released, a robust fibrin structure is essential [9,10].

## Conclusions

Dual benefits of PRF's releasing potential and MTA's sealing capacity are combined to speed up the irreversibly inflamed pulp tissue's healing process rather than extirpating it. Bioceramics, in comparison to calcium hydroxide, have demonstrated higher success rates due to their bioactivity, biocompatibility, low solubility, and ability to induce pulp proliferation. In addition to the preferred regenerative materials, other significant considerations that were taken into account when selecting cases for advanced endodontic treatment over traditional endodontics included patient motivation, compliance, oral hygiene habits, age, overall health, and diagnostic standards. Proper diagnosis and protocol adherence are crucial, and while hemostasis times vary across studies, immediate restoration ensures long-term treatment effectiveness.

This approach aims to preserve pulp vitality and has demonstrated high long-term success rates when performed under aseptic conditions with proper protocols for isolation, hemostasis, and clinical evaluation. Recent advances in microbiology, histology, and pulp capping materials have significantly improved outcomes, particularly with the use of bioceramic cement, which has shown superior bioactivity, biocompatibility, and antibacterial properties compared to traditional calcium hydroxide. Combining the dual benefits of PRF’s releasing potential and MTA’s sealing capacity can accelerate the healing of irreversibly inflamed pulp tissue, providing a viable alternative to extirpation. Long-term follow-up studies further support the efficacy of VPT, making it a viable and cost-effective option for maintaining dental health and function.
